# The role of Tks adaptor proteins in invadopodia formation, growth and metastasis of melanoma

**DOI:** 10.18632/oncotarget.12954

**Published:** 2016-10-27

**Authors:** Shinji Iizuka, Christopher Abdullah, Matthew D. Buschman, Begoña Diaz, Sara A. Courtneidge

**Affiliations:** ^1^ Sanford|Burnham|Prebys Medical Discovery Institute, La Jolla, CA, USA; ^2^ Department of Cell, Developmental & Cancer Biology, Oregon Health & Science University, Portland, OR, USA; ^3^ Biomedical Sciences Graduate Program, University of California, San Diego, La Jolla, CA, USA; ^4^ Department of Medicine, Division of Endocrinology and Metabolism, University of California, San Diego, La Jolla, CA, USA; ^5^ Los Angeles Biomedical Research Institute at Harbor-UCLA Medical Center, Torrance, CA, USA; ^6^ Department of Biomedical Engineering and Knight Cancer Institute, Oregon Health & Science University, Portland, OR, USA

**Keywords:** invadopodia, Tks5, Tks4, MT1-MMP, melanoma

## Abstract

Metastatic cancer cells are characterized by their ability to degrade and invade through extracellular matrix. We previously showed that the Tks adaptor proteins, Tks4 and Tks5, are required for invadopodia formation and/or function in Src-transformed fibroblasts and a number of human cancer cell types. In this study, we investigated the role of Tks adaptor proteins in melanoma cell invasion and metastasis. Knockdown of either Tks4 or Tks5 in both mouse and human melanoma cell lines resulted in a decreased ability to form invadopodia and degrade extracellular matrix. In addition, Tks-knockdown melanoma cells had decreased proliferation in a 3-dimensional type l collagen matrix, but not in 2-dimensional culture conditions. We also investigated the role of Tks proteins in melanoma progression *in vivo* using xenografts and experimental metastasis assays. Consistent with our *in vitro* results, reduction of Tks proteins markedly reduced subcutaneous melanoma growth as well as metastatic growth in the lung. We explored the clinical relevance of Tks protein expression in human melanoma specimens using a tissue microarray. Compared to non-malignant nevi, both Tks proteins were highly expressed in melanoma tissues. Moreover, metastatic melanoma cases showed higher expression of Tks5 than primary melanoma cases. Taken together, these findings suggest the importance of Tks adaptor proteins in melanoma growth and metastasis *in vivo*, likely via functional invadopodia formation.

## INTRODUCTION

Tumor cell metastasis is a complex, multistep process, in which tumor cells escape from the primary tumor and form secondary tumors at distant sites. Several steps involved in the metastatic cascade, including tumor dissemination, intra- and extravasation, as well as colonization and tumor growth at distant organs, require the cells to gain the ability to degrade and remodel the surrounding extracellular matrix (ECM) [1, 2].

Invadopodia have been described as one of the key regulators of cancer metastasis [3–5]. Invadopodia are actin-rich cell membrane protrusions that extend from the ventral surface of invasive cells in *in vitro* two-dimensional (2D) culture and display focal proteolytic activity towards the ECM [6, 7]. These cellular projections were first discovered in Src-transformed fibroblasts, where they were initially called podosomes [8], and were subsequently identified in a variety of invasive human cancer cells, where the term invadopodia was coined [9, 10]. Invadopodia are important regulators of protease-dependent cell invasion [11, 12].

A key regulator of invadopodia formation, the adaptor protein Tks5 (tyrosine kinase substrate with five SH3 domains - previously known as Fish), was originally discovered in our laboratory as a Src substrate [13–15]. Tks5 is encoded by the *SH3PXD2A* gene and contains a Phox-homology (PX) domain located at the N-terminus, five SH3 domains, as well as several polyproline motifs and two Src phosphorylation sites [16]. Tks5 plays a role in the function and formation of both podosomes and invadopodia [14, 16–20]. Our laboratory has demonstrated that Tks5 is required for mammalian development and cancer progression [6, 14, 19, 21–24]. We have also described the Tks4 (tyrosine kinase substrate with four SH3 domains) protein, a close homolog of Tks5, as a critical invadopodia component in Src-transformed fibroblasts [25], as well as a regulator of developmental processes [22, 26, 27]. Loss of Tks4 in Src-transformed fibroblasts resulted in the formation of pre-invadopodia structures, where many of the required structural and accessory proteins were appropriately localized, but ECM degradation did not take place [25]. However, Tks4 has not been studied in human cancer.

Cysteine, serine and metalloproteases (MMPs) are all found at invadopodia [6, 7]. Of all MMPs, MT1-MMP (also known as MMP14) appears to have the most significant role in cancer cell migration and invasion into the ECM [28, 29], likely through its diversity of substrates. MT1-MMP proteolytically activates other MMPs, such as MMP-2 and -13. It also directly cleaves many ECM components including type-I, -II and -III collagens, gelatin, fibronectin, fibrin, laminins 1 and 5, and vitronectin [30]. Regulation of MT1-MMP activity is thus a critical component of the invasive capacity of a cell. In particular, the subcellular localization of MT1-MMP plays an important role in regulating its function. MT1-MMP localization is controlled by its transmembrane domain and its 20 amino acid-long cytoplasmic tail. The cytoplasmic tail is critical for correct MT1-MMP localization and activity [31–33]. Once internalized, MT1-MMP can either be targeted for degradation or recycled back to the cell membrane [34, 35]. Thus, the surface expression and targeting of MT1-MMP to specific areas of the cell surface, particularly at invadopodia, represents a key mechanism for regulating its proteolytic activity. However, the regulation of cell surface targeting is incompletely understood [36].

Interestingly, there is also evidence that over-expressed MT1-MMP can promote growth in three-dimensional (3D) ECM [37], raising the possibility that the proteolytic properties of invadopodia might also be involved cancer cell growth. In keeping with this, our previous studies have suggested a role for Tks5 in growth in a more physiological 3D ECM context as well as *in vivo* [19, 21], in contrast to our previous findings that Tks5 was not required for cancer cell growth, which were based on studies performed in monolayer cell culture [14].

Our recent work has suggested the importance of Tks5 in cancer progression *in vivo* using a breast cancer orthotopic graft model [21]. Additionally, studies have demonstrated the clinical relevance of Tks5 expression in cancer, such as glial-derived brain tumors, lung adenocarcinomas, prostate cancer and breast cancer [21, 38–40]. However, the requirements for Tks adaptor proteins in melanoma are not well studied. Here, we explore the role of Tks4 and Tks5 in melanoma growth and metastasis and the clinical relevance of these proteins in human melanoma patient samples.

## RESULTS

### Tks adaptor proteins are required for functional invadopodia formation and mouse melanoma growth

**Figure 1:**
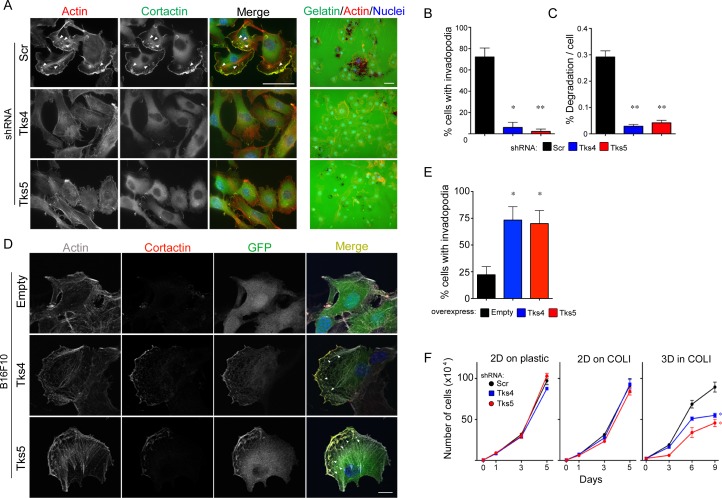
Invadopodia formation and function are required for mouse melanoma cell invasion and growth in 3D culture **A.** B16F10 cells with Scr, Tks4 or Tks5 knockdown were analyzed for invadopodia formation (left, grown on matrigel-coated coverslips) and ECM-degradation activity (right, grown on gelatin-coated coverslips). Cells were stained with F-actin (red) and cortactin (green) for invadopodia detection, and with Hoechst for detection of nuclei (blue). Representative invadopodia are highlighted with white arrows. Bars: 50μm. **B.**,**C.** Quantification of invadopodia formation and function. Percent of invadopodia-positive cells and gelatin degradation activity in B16F10 cells with Scr, Tks4 or Tks5 knockdown. *p < 5×10^−4^; **p < 10^−4^. **D.** B16F10 cells transfected with empty GFP (empty), Tks4-GFP (Tks4) or Tks5-GFP (Tks5) were analyzed for invadopodia formation. Invadopodia were visualized by F-actin (gray) and cortactin (red) on glass coverslips without matrigel coating). Nuclei were stained with Hoechst (blue). Representative invadopodia are highlighted with white arrowheads. Bars: 10μm. **E.** Quantification of invadopodia formation. Percent of invadopodia-positive cells was assessed. Data are presented as means ± SEM. *p < 0.05. **F.** Growth of B16F10 cells with Scr, Tks4 or Tks5 knockdown were analyzed in 2D conditions (plastic dish and type l collagen; 2D on COLl) and in 3D condition (3D in COLl). *p < 0.05, **p < 0.01. Data are presented as means ± SEM; for **B.** *n* = 9 of each graph; for **F.** *n* = 3. Statistical analysis for the growth curves was performed by comparing the AUC for each condition (Supplementary Figure 7).

Stylli et. al. have previously shown that invadopodia formation plays a role in mouse melanoma invasion *via* the Src-Tks5 pathway in cells overexpressing constitutively active Src and/or Tks5 [16]. However, the role of endogenous Tks5 in melanoma growth has not been studied. Moreover, the role of Tks4 in melanoma progression is not known. To investigate the roles of Tks adaptor proteins in melanoma, we began by using B16F10 cells (a highly metastatic murine melanoma cell line derived from spontaneously arising melanoma in C57BL/6 mice [41]) to generate cells that stably expressed shRNA specific for Tks4, Tks5 or a scrambled control sequence. Efficient knockdown of Tks4 or Tks5 was achieved (Supplementary Figure 1). In comparison to the scrambled shRNA-expressing control cells (72% invadopodia-positive), knockdown of either Tks4 or Tks5 in B16F10 cells resulted in a decrease in their ability to form invadopodia on Matrigel-coated glass coverslips (6% and 2% invadopodia-positive cells respectively), as judged by co-staining with actin and cortactin (Figure 1A, 1B). Consistent with this result, knockdown of each of the Tks adaptor proteins also decreased the degradation activity on a gelatin matrix (Figure 1A, 1C). To complement these experiments, we also over-expressed GFP-tagged forms of Tks4 or Tks5 into the B16F10 cells. In both cases, we observed co-localization of these scaffold proteins with cortactin (Figure 1D), as well as increased invadopodia formation when the cells were cultured on glass coverslips without added matrigel (Figure 1D, 1E). These data indicate that the expression level of Tks adaptor proteins modulate invadopodia formation in mouse melanoma cells.

It has previously been reported that cell proliferation in a 3D native (non-pepsin-extracted) type l collagen matrix required active MT1-MMP (in the case of cells engineered to overexpress the protease) and Tks5 (in the case of the endogenous protein in breast cancer cells) [21, 37]. We evaluated the requirement for Tks4 and Tks5 in mouse melanoma cells using this 3D collagen assay (Figure 1F). In comparison to scrambled control cells, knockdown of Tks4 or Tks5 in B16F10 cells resulted in an approximately 40% decrease (after 9 days in culture) in cell proliferation when embedded in a type l collagen matrix, but not when the cells were grown in 2D conditions (both on plastic dishes and on top of type l collagen) (Figure 1F). These data suggest that Tks adaptor proteins are also required for optimal 3D melanoma growth.

### Knockdown of Tks4 or Tks5 decreases the ability of B16F10 melanoma cells to colonize the lungs

**Figure 2:**
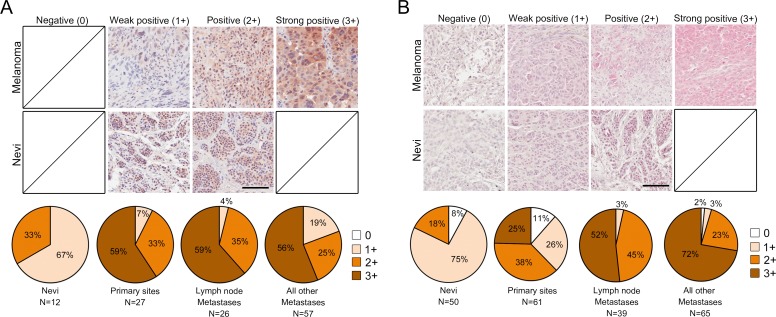
Tks adaptor proteins are required for melanoma metastasis *in vivo* C57Bl/6 mice were injected intravenously with 1 × 10^5^ B16F10 cells with Scr, Tks4 or Tks5 knockdown. After 28 days, the number of metastases per lung and size of each lung metastasis were evaluated. **A.** Representative macroscopic views of lung (top panels) and H&E-stained lung sections (middle and bottom panels). Arrowheads indicate metastatic melanoma in the lung, and high-magnification images (bottom panels) are shown from the indicated area in the middle panels. **B.** The number of lung metastases per lung and the size of each lung metastasis were determined by serial sections of each lung with H&E-staining. *p < 0.005, **p < 0.001, ***p < 10^−4^. Data are presented as means ± SEM; for (B, left) *n* = 10 for each group; for (B, right) *n* = Scr: 122, Tks4: 36, Tks5: 57. Scale bar: 1 mm (middle panel), 200μm (lower panel).

The use of the B16F10 cells allowed us to test whether Tks4 and Tks5 play a role *in vivo*, in immunocompetent animals. The scrambled and Tks adaptor protein knockdown cells were injected into the circulation through the tail vein, and the lungs were subsequently examined for tumor colonization, a so-called experimental metastasis assay. We also tried to examine tumor growth using a xenograft model; however, these data were difficult to analyze accurately because the highly invasive B16F10 cells disseminated from the primary site, and the resulting tumors could not be accurately measured or dissected from mice (not shown). Mice injected in the tail vein with the scrambled shRNA expressing melanoma cells showed considerable colonization of the lungs (Figure 2A), however there was a significant reduction in both lung tumor number and size in the Tks4 and Tks5 knockdown cells (Figure 2A, 2B). Taken together, these results suggest that Tks4 and Tks5 are important regulators of mouse melanoma growth *in vivo*.

### Tks adaptor proteins expression in human melanoma tissues

**Figure 3:**
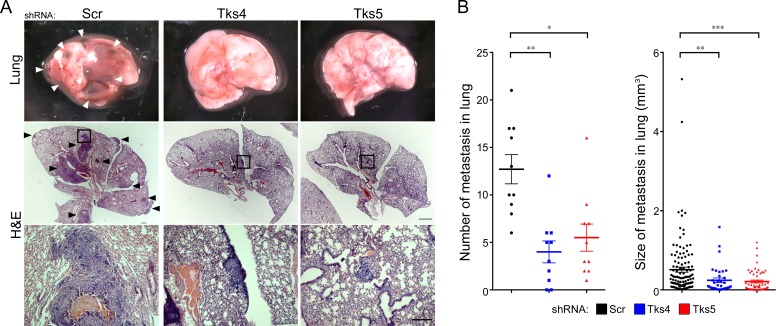
Tks adaptor proteins are highly expressed in human melanoma Tks4 **A.** and Tks5 **B.** IHC staining of human melanoma tissue microarray (*n* = 165 for Tks5, *n* = 110 for Tks4) including nevi as non-malignant control tissues (*n* = 50 for Tks5, *n* = 12 for Tks4). Representative images from melanoma cases and nevi are shown (top and middle panel). Scale bar 200μm. The staining intensity was scored as four levels as indicated in top panels. Distribution of IHC staining intensity for Tks4 and Tks5 in each melanoma stage is shown in the bottom pie graphs. Chi-squared analysis was performed to test statistical significance between progression and staining intensity (*p* < 0.05).

To investigate the potential clinical relevance of Tks4 and Tks5 expression in human melanoma, we performed immunohistochemical (IHC) analysis of tissue microarrays containing human melanoma specimens (total of 110 cases for Tks4 and 165 cases for Tks5 analysis). We used commercially available rabbit polyclonal Tks5 and Tks4 antibodies, which we had previously validated [21]. Each stained sample was blindly scored by a trained pathologist (SI) and representative images are shown in Figure 3A and 3B (top panels). Tissue microarray specimens also included nevi as a non-malignant tissue control (12 cases for Tks4 and 50 cases for Tks5 analysis). Nevi were predominantly weakly positive (1+), and there were no strong positives (3+) for either Tks4 or Tks5 expression. Compared to nevi samples, expression of Tks4 and Tks5 were largely positive (2+) or strongly positive (3+) in melanoma specimens. Interestingly, specimens with strong positive staining of Tks5 were highly increased in metastatic cases (lymph node metastasis: 52% and all other metastases: 72%) compared to primary melanoma cases (25%). A progression-dependent expression pattern was not found for Tks4, instead Tks4 expression was largely strong positive (3+) in all melanoma specimens. These analyses suggest that both expression of Tks4 and Tks5 were increased in human melanoma when compared to nevi (Figure 3A and 3B, bottom panels). These expression patterns were statistically significant using chi-squared analysis (*p* < 0.05). It has recently been reported that there are multiple isoforms of Tks5, generated by distinct promoters, and known as α, β and short [22, 39], and that it is only expression of the α form which correlates with outcome for lung and breast cancer [21, 39]. Currently available antibodies do not distinguish between the three isoforms. However, we could evaluate their expression in melanoma cell lines using isoform-specific qPCR and immunoblotting (supplementary Figure 2). We found that all but one cell line expressed Tks5α, at both the mRNA and protein level. The β isoform was generally not expressed. The short isoform was detectable at the mRNA level in all but one cell line, although interestingly protein expression was always low.

### Tks adaptor proteins are required for functional invadopodia formation and 3D growth in human melanoma cells

**Figure 4:**
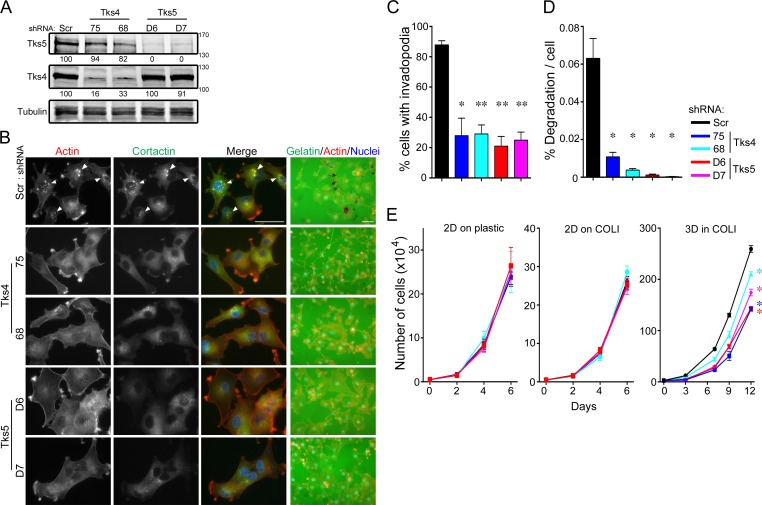
Tks adaptors are required for human melanoma invasion and growth in 3D culture conditions The human melanoma cell line, C8161.9 was infected with scrambled (Scr), Tks4- or Tks5-specific shRNA lentiviruses and analyzed in invadopodia formation and function assays. **A.** Lysates from C8161.9 cells infected with indicated shRNA viruses were immunoblotted with each antibody in the figure. **B.** C8161.9 cells with Scr, Tks4 or Tks5 knockdown were analyzed for invadopodia formation (left) and ECM-degradation activity (right, grown on gelatin coat coverslips). Invadopodia were visualized by F-actin (red) and cortactin (green). Nuclei were stained by Hoechst (blue). Representative invadopodia are highlighted with white arrowheads. Bars: 50μm. **C.**, **D.** Quantification of invadopodia formation and function. Percent of invadopodia-positive cells and gelatin degradation activity in C8161.9 cells with Scr, Tks4 or Tks5 knockdown. **E.** Growth of human melanoma cell lines, C8161.9 with Scr, Tks4 (75 and 68) or Tks5 (D6 and D7) knockdown were analyzed on 2D conditions (plastic dish and type l collagen; 2D on COLl) and on 3D condition (3D in COLl). p-value for **C.** *p < 0.005, **p < 10^−4^ (*n* = 9); for **D.** *p < 0.01 (*n* = 5). Statistical analyses were performed using Student's t test for **C.** and **D.**. Statistical analysis for the growth curves was performed by comparing the AUC for each condition (Supplementary Figure 7).

We next wanted to examine the role of Tks adaptor proteins in human melanoma. We previously demonstrated that knockdown of Tks5 in the human melanoma cell lines, C8161.9 and RPMI-7951, decreased melanoma cell invasion *in vitro* [14], but we did not quantify invadopodia at that time. There are no published reports on the role of Tks4 in any human cancer, except for the analysis of invadopodia function in one colon cancer cell line engineered to express constitutively active Src [42]. We first examined Tks4 and Tks5 localization in the human melanoma cell line C8161.9, and determined their localization to invadopodia (supplementary Figure 3). Tks4 or Tks5 were stably knocked down using two distinct shRNAs (shRNA-75 and -68 for Tks4, shRNA-D6 and D7 for Tks5) and then invadopodia formation and function evaluated in both C8161.9 (Figure 4) and WM793 (supplementary Figure 4). More than 70% of the cells formed invadopodia in control cells (88% in C8161.9 cells and 74% in WM793 cells), but knockdown of Tks4 or Tks5 led to significant decreases in invadopodia formation (less than 30% in each case). Consistent with these results, knockdown of Tks4 and Tks5 also significantly decreased the degradation of gelatin matrix. These data demonstrate that Tks adaptor proteins were required for functional invadopodia formation in human melanoma cells.

We next investigated the role of Tks proteins in human melanoma growth. Cells were placed in 3D matrices of native type l collagen, and growth compared to that in 2D (on plastic or on type l collagen) using both C8161.9 and WM793 cells with knockdown of Tks5 (D6 or D7) and Tks4 (75 and 68). Every 2-4 days, the total number of cells was counted, and the growth rates assessed (Figure 4E and Supplementary Figure 4). C8161.9 and WM793 cells with Tks4 or Tks5 knockdown proliferated normally under 2D culture conditions compared to scrambled controls (on plastic and on type l collagen). However, when these cell lines were cultured in 3D type l collagen, growth rates of Tks4- or Tks5-knockdown cells were significantly decreased compared to scrambled control cells. Interestingly, another invadopodia protein MT1-MMP, was also required for 3D, but not 2D growth of C8161.9 cells (supplementary Figure 5). These data indicate that Tks adaptor proteins, and likely functional invadopodia, were required for human melanoma growth in 3D conditions *in vitro*.

### Tks adaptor proteins are required for human melanoma progression *in vivo*

**Figure 5:**
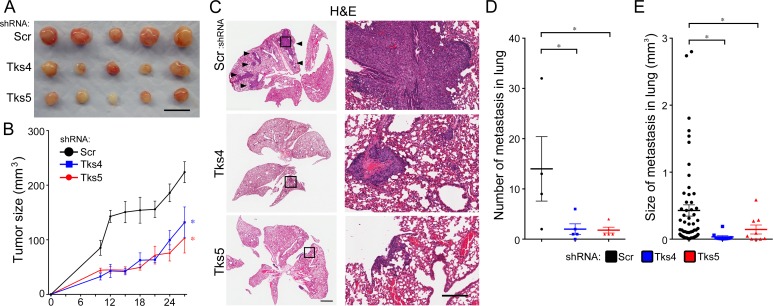
Tks adaptor proteins are required for human melanoma growth and metastasis *in vivo* For the tumor growth assay, nude mice were subcutaneously injected with 4 × 10^5^ C8161.9 cells with Scr, Tks4 (75) and Tks5 (D6) knockdown. **A.** Macroscopic view of all tumors (*n* = 5 in each group) is shown. Scale bar: 10 mm. **B.** Tumor volumes were measured every 2-3 days until 27 days after injection. *p < 0.05, **p < 0.01, ***p < 0.005, ****p < 5×10^−4^. Statistical analysis for the growth curves was performed by comparing the AUC for each condition (Supplementary Figure 7). For the metastasis assay, nude mice were intravenously injected with 1 × 10^5^ C8161.9 cells with Scr, Tks4 (75) and Tks5 (D6) knockdown. After 28 days, the lung metastases were evaluated. **C.** Representative H&E-stained lung sections are shown. Arrowheads indicate metastatic melanoma in the lung and high-magnification images (right panels) are shown from the indicated area in left panels. Scale bar: 1 mm (left panel), 200 mm (right panel). The number of lung metastases per lung **D.** and size of each lung metastasis **E.** were evaluated by serial sections of each lung with H&E-staining. One of the mice in the control group (scrambled shRNA) died during the experiments. p-values for **D.** *p < 0.05; for **E.** *p < 0.05. Data are presented as means ± SEM; for **B.** *n* = 5 for each group; for **D.** *n* = Scr: 4, Tks4 and Tks5: 5; for **E.** *n* = Scr: 56, Tks4: 10, Tks5: 9.

We next conducted xenograft tumor growth assays and experimental metastasis assays *in vivo* (Figure 5). We first tested the appropriateness of C8161.9 and WM793 cell lines for *in vivo* experiments. C8161.9 and WM793 cells were subcutaneously injected into nude mice, and the size of tumor was measured every 2-3 days. In addition, these cell lines were also introduced *via* the tail vein to test metastatic ability. We noted that C8161.9 cells grew well *in vivo* both in the primary site and in the lung, but no tumor growth at either primary site or lungs was observed with WM793 cells (data not shown). Therefore we used C8161.9 cells to test the role of Tks adaptor proteins *in vivo*. Melanoma cells with knockdown of either Tks4 or Tks5 showed significant reduction of primary tumor size compared to controls (panels A, B). Consistent with our previous results using mouse melanoma cells, Tks knockdown in human melanoma cells also led to significant decrease in both the number and the size of metastases in lungs after tail vein injection (panels C-E). Taken together, these results indicate that both Tks4 and Tks5 are required for human melanoma growth and experimental metastasis.

### Tks adaptor proteins regulate MT1-MMP cell surface expression

**Figure 6:**
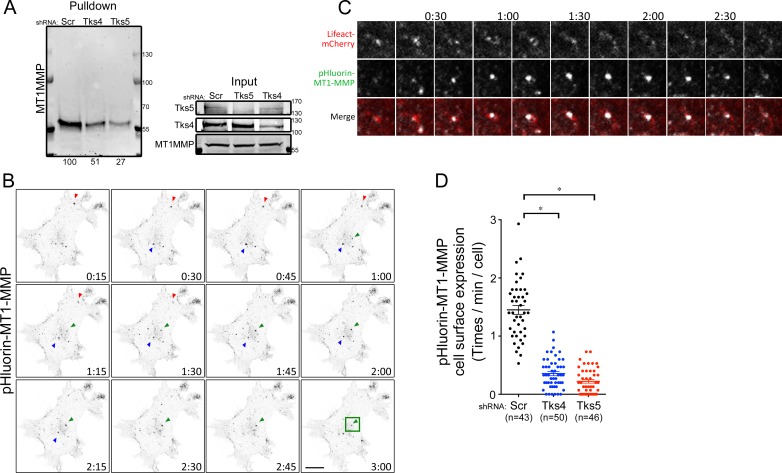
Tks adaptor proteins regulate surface expression of MT1-MMP **A.** Cell surface expression of MT1-MMP in C8161.9 cells with Scr, Tks4 or Tks5 knockdown. Cells were labeled with biotin, and cell surface proteins were pulled down, using streptavidin-agarose beads and blotted by MT1-MMP antibody. Total expression levels of each protein were shown as loading controls. **B.** Time-lapse images (15 seconds per frame) of pHluorin expression at cell surface in C8161.9 with scrambled shRNA. Three representative GFP flashes were indicated by arrowheads with different color (Red, Green and Blue). **C.** High magnification images with Lifeact-mCherry (Red) and pHluorin-MT1-MMP (Green) from the green square shown in **B.**. **D.** Quantification of exocytic event (cell surface expression of MT1-MMP). n, total number of cells analyzed for each stable cell line. Data are presented as means ± SEM.

MT1-MMP is known to be recruited to invadopodia and contributes to matrix degradation [28, 43, 44]. And our laboratory has previously described that Tks4 is required for MT1-MMP localization to invadopodia in Src-transformed fibroblasts [25]. In keeping with this, we found that MT1-MMP co-localized with GFP-tagged Tks4, and to a lesser extent Tks5, in the human melanoma cell line C8161.9 (supplementary Figure 6). To assess whether Tks adaptor proteins regulate the cell surface expression of MT1-MMP in melanoma cells, we used two experimental approaches. First, the expression level of MT1-MMP at the cell surface was examined by a cell surface biotinylation pulldown assay (Figure 6A). Although knockdown of Tks proteins had no effect on the total cellular levels of MT1-MMP, cell surface expression of MT1-MMP was reduced in Tks protein knockdown cells (49% reduction in Tks4 knockdown, 73% reduction in Tks5 knockdown). Second, these findings were confirmed by analyzing MT1-MMP exocytic events using pHluorin tagged MT1-MMP. We also overexpressed Lifeact-mCherry to visualize actin structures in these cells. The pHluorin-tag is a pH-dependent green fluorescent protein (GFP) variant that is fluorescent at neutral pH (extracellular region), but not at acidic pH (endosomal vesicle) [45], and has been used to study MT1-MMP trafficking [46]. For these experiments, pHluorin-MT1-MMP and Lifeact-mCherry overexpressing C8161.9 cells were infected with scrambled, shRNA-Tks4 and shRNA-Tks5. The actin structures visualized by Lifeact-mCherry were used for focusing on the ventral surface of the cell, and the GFP flash at the cell surface was analyzed by time-lapse confocal microscopy to visualize exocytic events (cell surface expression) of MT1-MMP (Figure 6B-6D). Consistent with our findings in the pulldown assay (Figure 6A), MT1-MMP exocytic events in the Tks4 and Tks5 knockdown cells were significantly reduced compared to control cells suggesting that both Tks4 and Tks5 are necessary for MT1-MMP surface expression, likely by regulating its exocytosis.

## DISCUSSION

Previous findings have indicated that the adaptor protein Tks5 regulates melanoma invasiveness *in vitro* [14, 16]. However, the role of Tks4 in cancer cells has not previously been addressed. In the present study, we demonstrated that both Tks4 and Tks5 were required for functional invadopodia formation, in both mouse and human melanoma cells. We previously reported that Tks4 is localized at invadopodia in Src-transformed fibroblasts, and that knockdown of Tks4 led to incomplete invadopodia formation as defined by reduced polymerized actin, lack of ECM degradation, and failure to accumulate MT1-MMP [25]. Overexpression of Tks5 rescued the actin polymerization at invadopodia, but not ECM degradation or MT1-MMP targeting [25]. We show here that in melanoma cells, both Tks4 and Tks5 were required for invadopodia formation and function, suggesting that they have non-overlapping functions in this cell type. Alternatively, it is also possible that Tks4 and Tks5 have similar functions, and that the total dosage of these adaptor proteins is important. In this scenario, knockdown of either Tks4 or Tks5 would reduce the level of the adaptors below a critical threshold required for invadopodia formation. In keeping with this latter hypothesis, Tks4 knockout MEFs started to re-form functional invadopodia structures *via* up-regulation of Tks5 after prolonged passage in culture, or upon overexpression of Tks5 [25]. Furthermore, it has not proven possible to simultaneously knock down both Tks4 and Tks5 (unpublished). Further studies into the binding partners and regulation of the Tks proteins are required to fully understand their distinct and possibly overlapping roles.

What is the mechanism by which the Tks adaptors, and invadopodia, regulate invasive capacity? It is known that MT1-MMP is one of the key proteases required for degradation of the ECM surrounding cancer tissue [47], and that MT1-MMP localizes to invadopodia [48]. The trafficking of MT1-MMP is a highly regulated process [28], involving, for example, an exocytic mechanism connecting MT1-MMP positive late endosomes and the plasma membrane, which requires ARF6 and JIP3/4 [49, 50]. We show here that reduced exocytosis of MT1-MMP vesicles following Tks adaptor knockdown accounted for the loss of surface expression of MT1-MMP. It is possible that loss of invadopodia caused a concomitant loss of exocyst docking sites, although future studies will be necessary to understand fully the mechanism.

High expression of Tks5 has previously been reported in some cancer types, such as glial-derived brain tumors [38], lung adenocarcinomas [39], prostate cancer [40] and breast cancer [21], and can correlate with poorer outcome [21, 38, 39]. Here, we show that high expression of Tks5, as well as Tks4, was also present in human melanoma samples. Moreover, higher expression levels of Tks5 were more frequent in metastatic melanoma cases, compared to primary tumors, although this was not observed for Tks4. These findings prompted us to evaluate the roles of these adaptor proteins in melanoma progression and metastasis. Using an experimental metastasis assay, and both a mouse and a human melanoma cell line, we found fewer tumors in the lungs of mice injected with Tks4 or Tks5 knockdown cells, compared to the scrambled controls. This is consistent with a recent report that the Tks adaptors, and invadopodia, are required for extravasation in experimental model systems [4]. However, we also noticed that those metastases that did form in the lungs of mice injected with knockdown cells were smaller than controls, suggesting that Tks adaptors were also required for efficient tumor growth. In keeping with this, C8161.9 also grew more poorly as subcutaneous tumors in the absence of Tks4 or Tks5. In previous studies we noted reduced growth of Src-transformed fibroblasts and human breast cancer cells in subcutaneous and orthotopic sites respectively, when Tks5 expression levels were reduced [19, 21]. Importantly, our use of the B16F10 cells introduced syngeneically into C57Bl6 mice has allowed us to rule out the possibility that these findings only apply to immunocompromised mice. It has previously been shown that tyrosine phosphorylation of Tks5 is required for invadopodia formation in melanoma cells [16], however whether this phosphorylation is catalyzed by a Src family kinase is as yet unexplored.

We have also shown both here and in previous work using a breast cancer cell line [21] that decreased levels of Tks adaptor proteins resulted in reduced growth in 3D-ECM culture conditions. It is formally possible that Tks4 and Tks5 have other functions, separate from their roles in invadopodia formation, which are necessary for tumor cell growth in 3D *in vitro* and *in vivo*. However, we note that other invadopodia proteins, for example MT1-MMP (supplementary Figure 5 and [37]), Cdk5 [51] and cortactin [52], are also necessary for 3D and/or *in vivo* growth in pancreatic cancer and head-and-neck squamous cell cancers. Collectively, these data suggest that the prior almost exclusive use of 2D culture systems has led us to overlook a role for invadopodia in cancer cell growth. How 3D growth might be facilitated by invadopodia is not yet clear, although proteolysis is certainly likely to be involved. One possibility is that the proteolytic activity of these structures is required to create physical space to grow. Alternatively, growth factor and cytokine processing by proteases, including of the MMP class [53], may be necessary to overcome the restrictions in place in 3D environments. We are currently investigating these possibilities.

In summary, our findings indicate that both Tks4 and Tks5 have essential roles for melanoma growth and metastasis *in vivo*. Our data suggest a model where, through the Tks adaptor proteins, invadopodia formation and surface recruitment of MT1-MMP drive tumor growth and metastasis. Moreover, Tks adaptor proteins are highly expressed in melanoma patient samples. Although the full molecular mechanism of invadopodia-mediated cancer progression remains to be elucidated, we speculate that invadopodia inhibitors might have potential as therapeutic agents to prevent cancer progression.

## MATERIALS AND METHODS

### Cell lines

The C8161.9 human melanoma cell line was purchased from ATCC. The WM793 human melanoma cell line was a gift from Dr. Gary G. Chiang (Abbvie, Chicago, IL). The B16F10 mouse melanoma cell was a gift from Dr. Peter Lock (La Trobe University, Melbourne, Australia). Cells were grown in DMEM (Mediatech, Manassas, VA, USA) containing 10% fetal bovine serum (FBS). FBS was obtained from HyClone. All experiments were performed in complete medium in the absence of antibiotics.

### Reagents and antibodies for immunoblotting and immunohistochemistry

The antibody for Tks5 (anti-Tks5 1737) was generated by the Courtneidge laboratory and used for immunoblotting and immunofluorescence experiments (Lock et al., 1998) and Tks5 antibody (ProteinTech) was used for immunohistochemistry, as previously described (Blouw et al., 2015). The anti-Tks4 antibody (09-267) was previously described (Buschman et al., 2009) and is available from EMD Millipore (Temecula, CA, USA). The following commercial antibodies were used: anti-cortactin (4F10) antibody (EMD Millipore), anti-γ-tubulin antibody (Sigma-Aldrich, St. Louis, MO, USA), MT1-MMP antibody (Abcam or EMD Millipore). Alexa Fluor 568-conjugated phalloidin (1:500 in PBS containing 0.5% BSA and 0.1% Triton X-100; EMD Chemicals Inc., Darmstadt, Germany) was used for actin staining. For secondary antibodies, Alexa Fluor 680 goat anti-rabbit IgG (Invitrogen, Carlsbad, CA, USA) or IR800 (Rockland Immunochemicals, Gilbertsville, PA, USA) were used for immunoblotting, and Alexa Fluor-594-conjugated or -488-conjugated antibodies (Chemicon, Temecula, CA, USA) were used for immunohistochemistry. For gelatin degradation assays, Oregon green gelatin (0.2 mg/ml in PBS containing 2% sucrose; Invitrogen) was used.

### Immunoblotting

Cell lysates were prepared by washing cells twice with cold Tris-buffered saline (TBS) containing 100 μM Na_3_VO_4_ and then lysing in 50 mM Tris-HCl (pH 7.5), 250 mM NaCl, 1% Triton X-100, 50 mM NaF, 100 μM Na_3_VO_4_ and 1mM EDTA lysis buffer containing a dissolved complete Mini protease inhibitor tab (Roche Diagnostics, Germany). Supernatant of cell lysates was assayed for total protein content using the BCA protein assay (Thermo Fisher Scientific, Rockford, IL), and 70 μg of total protein per sample was separated in a 7.5% polyacrylamide gel (Invitrogen). Secondary antibodies were conjugated to Alexa Fluor 680 or IR800, and membranes were scanned using an infrared imaging system (Odyssey; LI-COR Biosciences, Lincoln, NE).

### Biotin labeling of Cells

Biotin labeling of cell surface proteins was performed as previously described [35]. Briefly, cells were washed twice in ice-cold PBS and incubated in PBS for 10 min at 4°C. Cells were then labeled with biotin by incubating cells at 4°C with 0.5 mg/ml NHS-SS-Biotin (Thermo Fisher Scientific) for 30 min. Biotinylated cells were lysed in cell lysis buffer, and labeled cell surface proteins were precipitated with streptavidin-agarose beads (Thermo Fisher Scientific).

### MT1-MMP-pHluorin

C8161.9 cells stably expressing MT1-MMP-pHluorin and Lifeact-mCherry were infected with the indicated shRNAs (scrambled, Tks5 and Tks4), and stable pools of cells were established. The cells were plated on glass bottomed dishes (MatTek, MatTek Corporation) and incubated for 6 hr before imaging. The cells were imaged on a microscope (Zeiss LSM 880 laser-scanning confocal microscope with AiryScan, 4 images/min, total 15 min). The number of exocytic events of MT1-MMP-pHluorin was manually counted and measured per minute and per cell.

### Invadopodia staining and gelatin degradation assay

Invadopodia staining and gelatin degradation assays were performed as previously described [54]. Briefly, cells were grown on glass coverslips with or without Matrigel-coating (invadopodia formation assay) or gelatin-coated coverslips (degradation assay) and fixed with 4% paraformaldehyde/PBS (Electron Microscopy Sciences). For the invadopodia assay on Matrigel-coated coverslips, BD Matrigel Basement Membrane Matrix (BD Biosciences, Bedford, MA, USA) was prepared according to the manufacturer's instructions. Briefly, Matrigel was diluted by serum-free medium (1:10 dilution) on ice and added to coverslips for 1 hr at RT. After rinsing with serum-free medium, B16F10 cells were cultured on the coverslips and experiments performed. After permeabilization with 0.1% Triton X-100/PBS for 15 min, the cells are incubated with primary antibodies overnight at 4°C. Cells were washed and incubated with Alexa Fluor-conjugated secondary antibodies and phalloidin. Fluorescence microscopy images were obtained with a fluorescent microscope (Axioplan2; Carl Zeiss) equipped with a charge-coupled device camera (AxioCam HRm; Carl Zeiss) and AxioVision software (Carl Zeiss). For each invadopodia experiment, the number of cells forming invadopodia was quantified in 5-9 microscope fields (63x) imaged randomly and percentage of invadopodia forming cells was assessed. For each ECM degradation experiment, 5-9 microscope fields (40x) imaged randomly. The percentage of degraded area was quantified with ImageJ software (National Institutes of Health) and normalized to the number of nuclei in that area was represented as “% degradation per cell”.

### Constructs

pLKO.1 shRNA lentiviral plasmids used for scrambled and human Tks5 knockdown were purchased from Sigma-Aldrich. The clones used were TRCN0000136014 (referred to as clone D6) and TRCN0000136512 (referred to as clone D7). Mission shRNA (pLKO.1) constructs against mouse and human Tks4, were obtained from Sigma-Aldrich. The clones used were TRCN0000147775 (referred to as clone #75) and TRCN0000129568 (referred to as clone #68). MT1-MMP-pHluorin Lentivirus vector was kindly provided by Dr. Philippe Chavrier (Institut Curie, Paris). pEGFP-N1 DNA plasmids used for overexpression experiments were purchased from Clontech (Mountain View, CA). Human Tks4 or Tks5 was overexpressed using Lipofectamine 2000 from Invitrogen (Carlsbad, CA, USA) in either B16F10 or C8161.9 cells according to the manufacturer's protocol. Two days post-transfection, cells were visualized for invadopodia formation.

Lentiviral preparations were made by the viral core facilities at the Sanford|Burnham|Prebys Medical Discovery Institute (La Jolla, CA) and Oregon National Primate Research Center (Molecular & Cell Biology, Lentivirus Service) at the Oregon Health and Science University (Portland, OR).

### 3D proliferation assay

Type l collagen 3D cultures were performed as described previously [37]. Briefly, rat tail type l collagen (BD Biosciences, Bedford, MA, USA) was prepared to a final concentration of 2 mg/ml, and 5,000 to 25,000 cells were added to the collagen mix before gelling. Spread cells were grown for 9-14 days in DMEM containing 10% FBS. The matrix was dissolved with 2 mg/ml collagenase (Worthington, NJ, USA) and cell numbers were determined by hemocytometry.

Type l collagen 2D cultures were performed according to the manufacturer's instructions. Briefly, rat tail type l collagen (BD Biosciences) was prepared at a final concentration of 50μg/ml in 0.02M acetic acid and incubated with coverslips at room temperature for 1 h. After aspirating the remaining solution, it was rinsed well with PBS to remove any remaining acid. 5,000 to 10,000 cells were added and cultured for 5-6 days in DMEM containing 10% FBS, and cell numbers were counted. The proliferation assay on plastic plates was performed by the same method described previously without type l collagen.

### Subcutaneous tumor growth and experimental metastasis assays

All animal experiments were conducted in accordance with the NIH Guide for the Care and Use of Laboratory Animals. Subcutaneous implantation was carried out with minor modifications as described previously [19]. In short, cells were harvested by trypsinization and resuspended in PBS/Matrigel mixture (1:1 ratio) to a final concentration of 4×10^6^ cells/ml. Athymic nude mice (Harlan, Indianapolis, Indiana) were injected in the flank with 100 μl, and tumors were allowed to form for 27 days. Tumor growth was measured every 2-3 days using calipers. The longest (L) and shortest (S) measurements were recorded, and tumor volumes were calculated as Volume = 0.5*(L x S^2^) and expressed as mean volume ± SEM.

For the lung metastasis assay, cells were collected as described above and resuspended at a final concentration of 5 × 10^6^ cells/ml in PBS. Of this suspension, 100 μl (total 5 × 10^5^ cells/mouse) was injected into the tail veins of athymic nude mice (for human cell line) and C57BL/6 (for mouse cell line). After 28 days, the mice were sacrificed, and the lungs were dissected out. The number and size of metastases were assessed with minor modifications as described in previously [19]. Briefly, to determine the size and number of metastases, images from H&E-stained serial sectioning samples were taken by inverted TE300 Nikon Microscope with Spot RT Acquisition and Processing Software or Aperio ScanScope CS Slide Scanner, and the metastatic tumor size was quantified using ImageJ software or Aperio software. Numbers of metastases in lung were counted from H&E-stained serial sectioning samples.

### Tissue microarray and scoring scheme

Tks4 and Tks5 antibodies were used to stain melanoma tissue microarray samples obtained from the Cancer Diagnosis Program (CDP) of the National Cancer Institute following approval from the National Disease Research Interchange (NDRI) (http://ndriresource.org/). Antigen retrieval was performed according to the manufacturer's guidelines using Antigen Retrieval Buffer (DAKO S1699). After incubation with Tks4 or Tks5 antibody, the sections were incubated with secondary antibody using the manufacturer's guidelines of the MACH2 Rabbit AP-Polymer (Biocare Medical) and visualized with either Vulcan Fast Red Chromogen (Biocare Medical, pink) or DAB (brown). Sections were counterstained with hematoxylin. A scoring system, developed by a trained pathologist (SI), of 0, no staining; 1+, low staining or staining in < 25% of tumor cells (weak positive staining); 2+, moderate staining (positive staining); and 3+, high staining (strong positive staining). All slides were scored blindly.

### Statistical analysis

Statistical significance was determined by calculating the p-value (P) using the paired Student's *t* test. *p* < 0.05 was considered to be statistically significant. The numbers of samples (n) are indicated in each figure legend. For the immunohistochemical scoring analysis, statistical significance was determined by using the chi-squared significance test (*p* < 0.05 is significant). For 2D, 3D, and tumor growth curves, area under the curve analysis was performed on the individual growth curves using the Area Under the Curve (AUC) function in the GraphPad Prism software. Means and SEM were then calculated and Student's *t* test was used to determine significance. AUC graphs can be found in Supplementary Figure 7.

### RNA isolation and qPCR analysis

RNA was isolated from human melanoma cell lines using the RNeasy RNA isolation kit (Qiagen). cDNA was generated using the SuperScript III First-Strand Synthesis kit (Invitrogen). Absolute expression analysis was done by using standard curve for each primer sets that made by DNA plasmids containing each isoforms as template, and the gene copy number was normalized by housekeeping gene (RPLP0) to compare the expression level of each isoforms between cell lines (copy number index). Primers for each isoform of Tks5 were generated against unique regions for the alpha, beta, and short isoforms and can be found in Table 1.

**Table 1:** Primer sets for human Tks5 isoforms and housekeeping gene

hTks5 alpha-F:	TAATCAATGTGACCTGGTCTG
hTks5 alpha-R:	TTGGGGTCCTTCTGGCCAC
hTks5 beta-F:	TGTCTATCTGTTGTTGCTTCTTTTTC
hTks5 beta-R:	CAGAAGTGCTCACGAACCAC
hTks5 short-F:	TGGCTCACCGCGTGCTTTCTG
hTks5 short-R:	CAGAAGTGCTCACGAACCAC (same as beta-R)
hRPLP0-F:	GGCGACCTGGAAGTCCAACT
hRPLP0-R:	CCATCAGCACCACAGCCTTC

## SUPPLEMENTARY MATERIAL


