# Supplementary material for: The role of Tks adaptor proteins in invadopodia formation, growth and metastasis of melanoma
Source: Oncotarget. 2016 Oct 27;7(48):78473–86. doi: 10.18632/oncotarget.12954 (PMC5346654; doi:10.18632/oncotarget.12954)
Supplement: Supplementary file 1 [file oncotarget-07-78473-s001.pdf]

The role of Tks adaptor proteins in invadopodia formation, growth and metastasis of melanoma

Supplementary Material

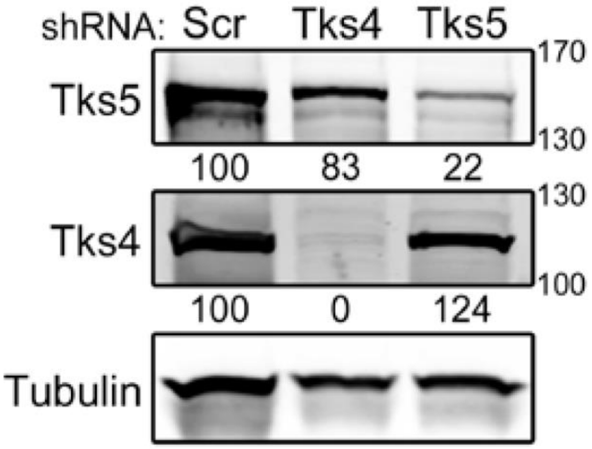

Supplemental Figure 1. Knockdown of Tks adaptor proteins analyzed by immunoblot.
Lysates from B16F10 cells infected with scrambled (Scr), Tks4- or Tks5-specific shRNA viruses were immunoblotted with the indicated antibodies. Tubulin is shown as a loading control.

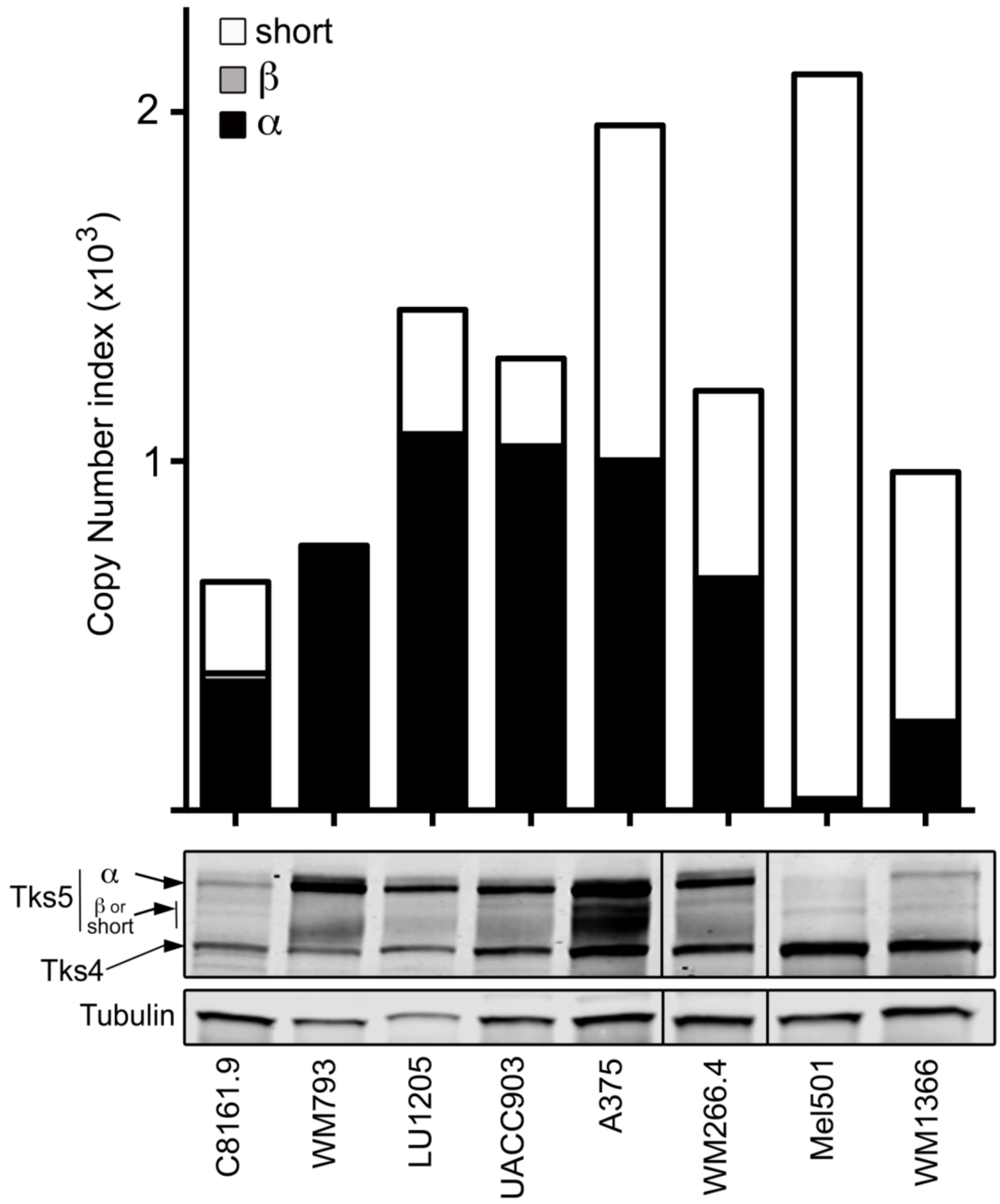

Supplemental Figure 2. Expression of Tks5 isoforms in human melanoma cell.
Expression of Tks5 isoforms was analyzed by qPCR and immunoblotting from each of the panel of human melanoma cell lines. Primer sets unique to each of Tks5 isoforms are shown in Table1.

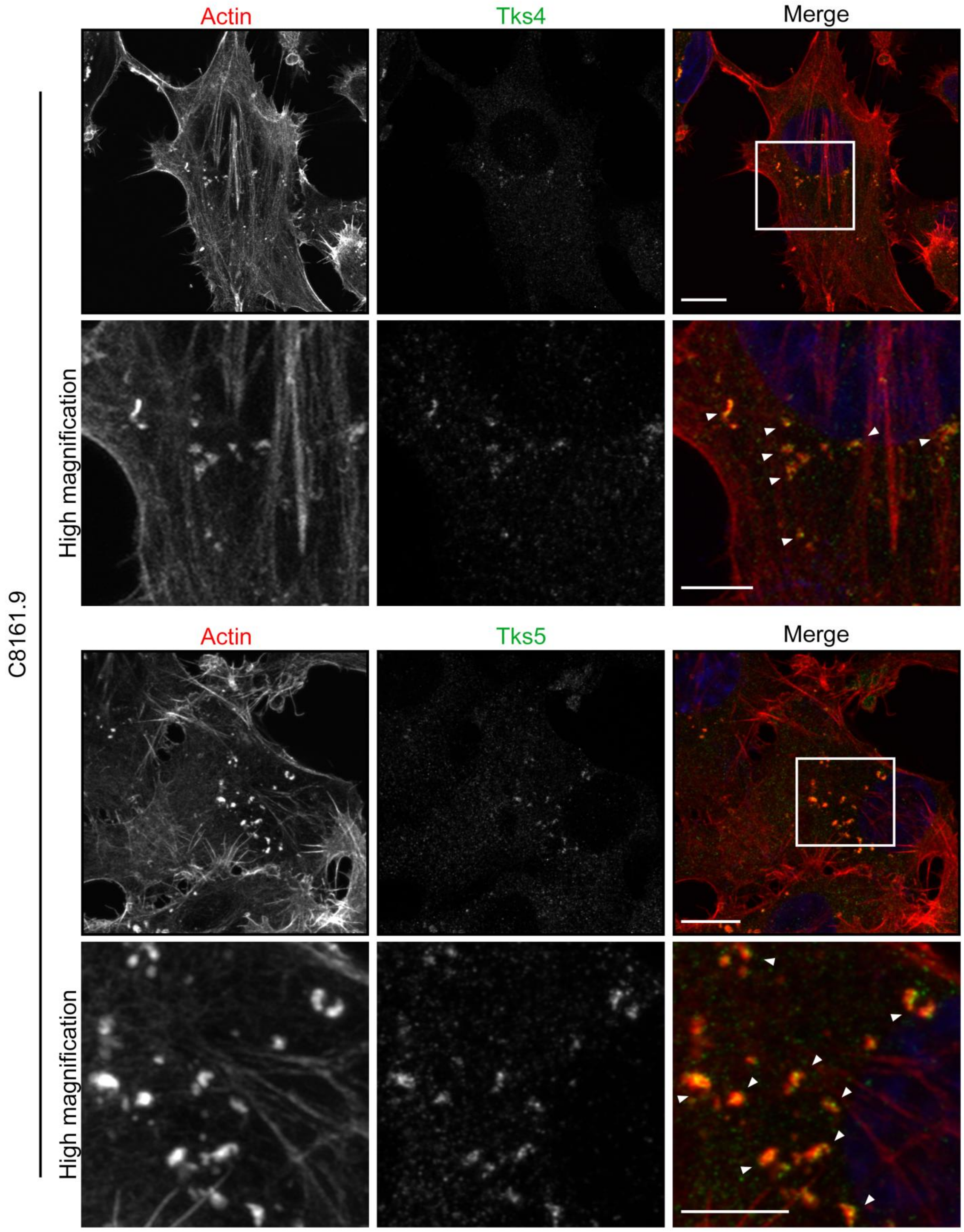

Supplemental Figure 3. Tks adaptors localized at invadopodia in human melanoma cells.
C8161.9 cells were stained for F-actin (red), Tks4 or Tks5 (green) and nuclei (blue) to visualize endogenous Tks adaptor protein localization. Representative invadopodia that co-localized with actin and Tks adaptors were highlighted with white arrowheads. High magnification images from white squares are shown in lower panel. Bars: 10⌠m (top panel) and 5⌠m (high magnification images in lower panel).

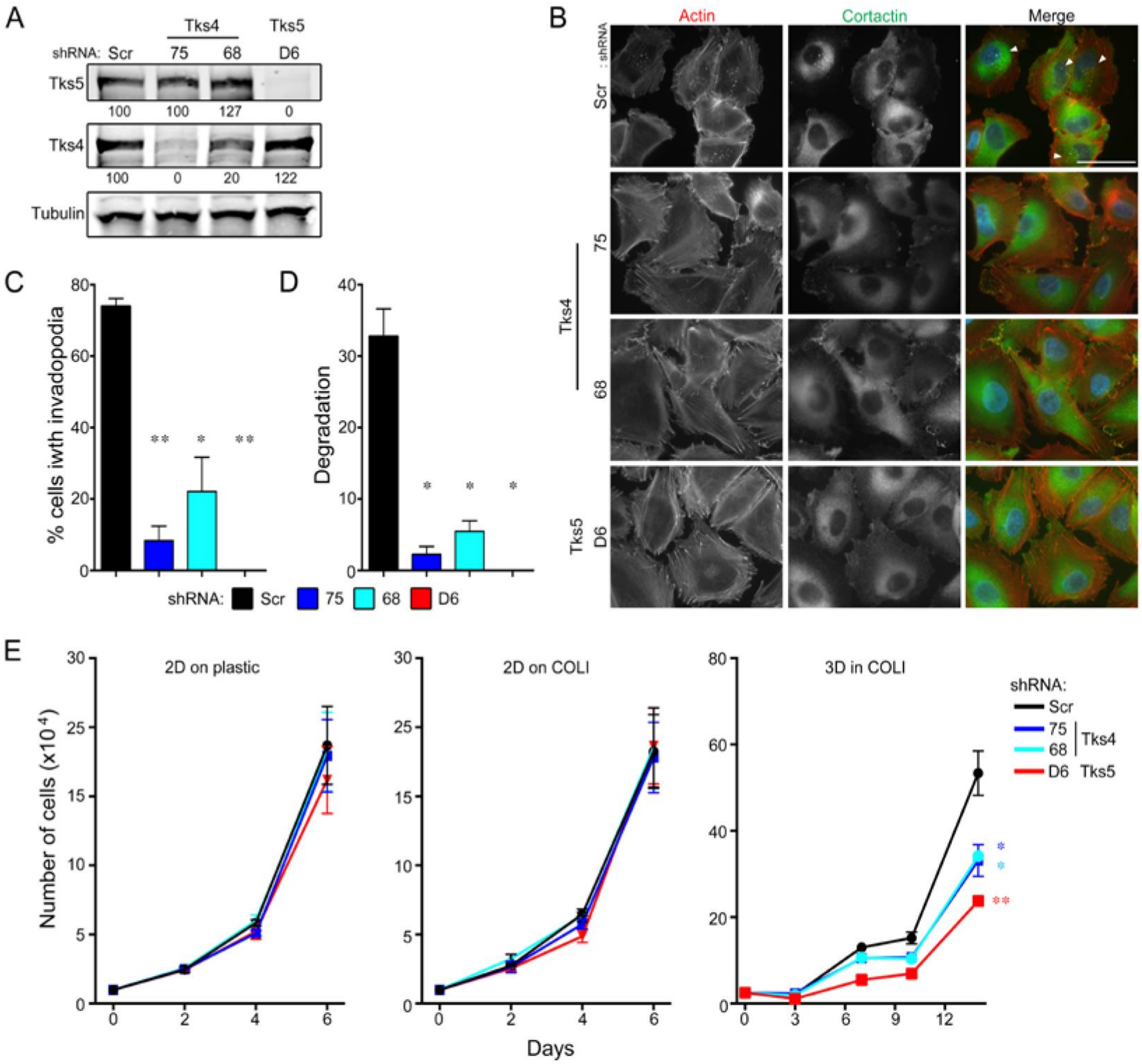

Supplemental Figure 4. Tks adaptors are required for human melanoma invasion and growth in 3D culture conditions.

(A) WM793 cells infected with scrambled (Scr), Tks4 (75 and 68)- or Tks5 (D6)-specific shRNA viruses were immunoblotted with the indicated antibodies. Expression levels of Tks4 and Tks5 were evaluated (corrected for tubulin expression) and relative expression levels are shown below each lane. (B) WM793 cells with Scr, Tks4 or Tks5 knockdown were analyzed for invadopodia formation (left) and ECM-degradation activity (right, grown on gelatin-coated coverslips). Invadopodia were visualized by F-actin (red) and cortactin (green). Nuclei were stained with Hoechst (blue). Representative invadopodia are highlighted with white arrowheads. Bars: 50μm. (C, D) Percent of invadopodiapositive cells and of cells with gelatin degradation under invadopodia in WM793 cells with Scr, Tks4 or Tks5 knockdown. (E) WM793 with Scr, Tks4 (75 and 68) or Tks5 (D6) knockdown were analyzed in 2D (plastic dish and type I collagen; 2D on COLI) and in 3D conditions (3D in COLI). p-value for (C) $*p<0.005$, $**p<0.001$ (n=5); for (D) $*p<0.01$ (n=5). Data are presented as means ± SEM. Statistical analysis for the growth curves was performed by comparing the AUC for each condition (Supplemental Figure 7).

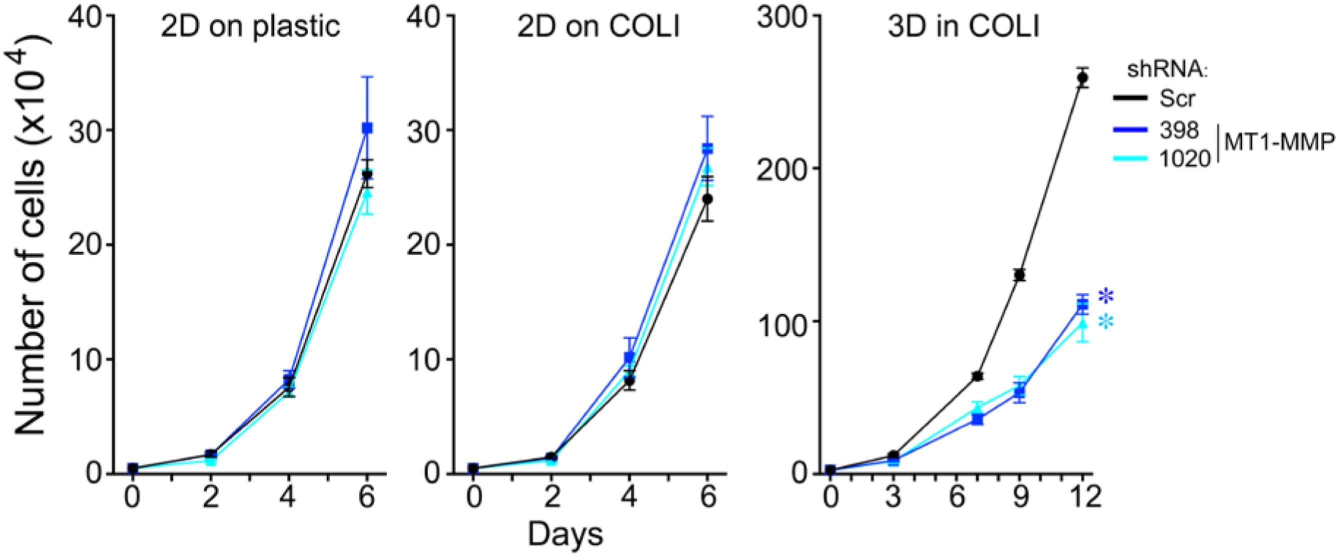

Supplemental Figure 5. MT1-MMP is required for human melanoma growth in 3D culture conditions. Growth of human melanoma cell lines, C8161.9 with Scr and MT1MMP (398 and 1020) knockdown were analyzed on 2D conditions (plastic dish and type I collagen; 2D on COLI) and on 3D condition (3D in COLI). p-value *p<0.005. Statistical analysis was performed by comparing the AUC for each condition (supplemental Figure 7).

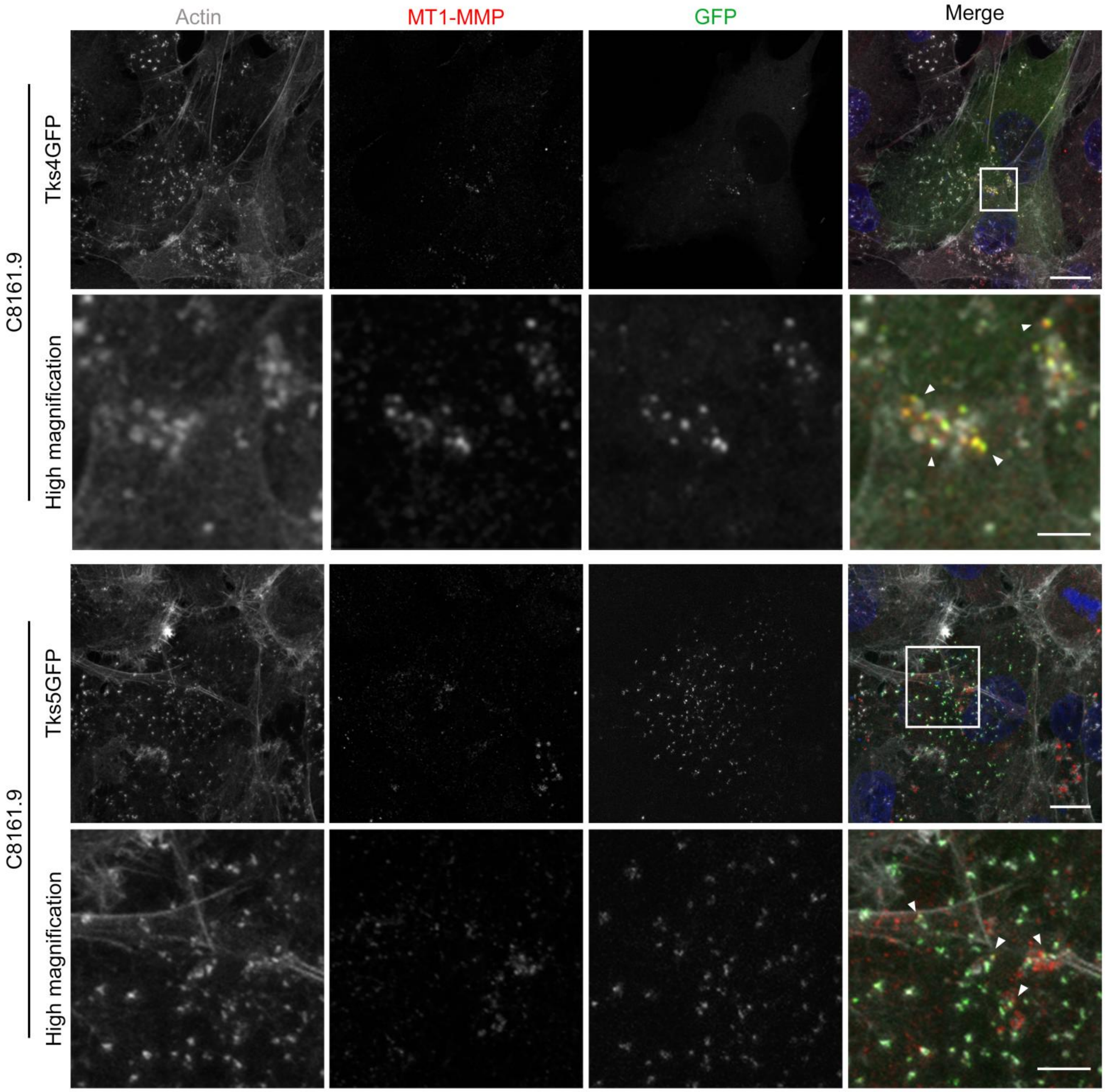

Supplemental Figure 6. Tks adaptors localized with MT1-MMP at invadopodia in human melanoma cells.
C8161.9 cells transfected with empty GFP (empty), Tks4-GFP (Tks4) or Tks5-GFP (Tks5) were stained for F-actin (gray), MT1-MMP (red) and nuclei (blue). Representative invadopodia that co-localized with actin, MT1-MMP and Tks adaptors (green) were highlighted with white arrowheads. High magnification images from white squares are shown in lower panel. Bars: 10μm (top panel) and 2μm (high magnification images in lower panel).

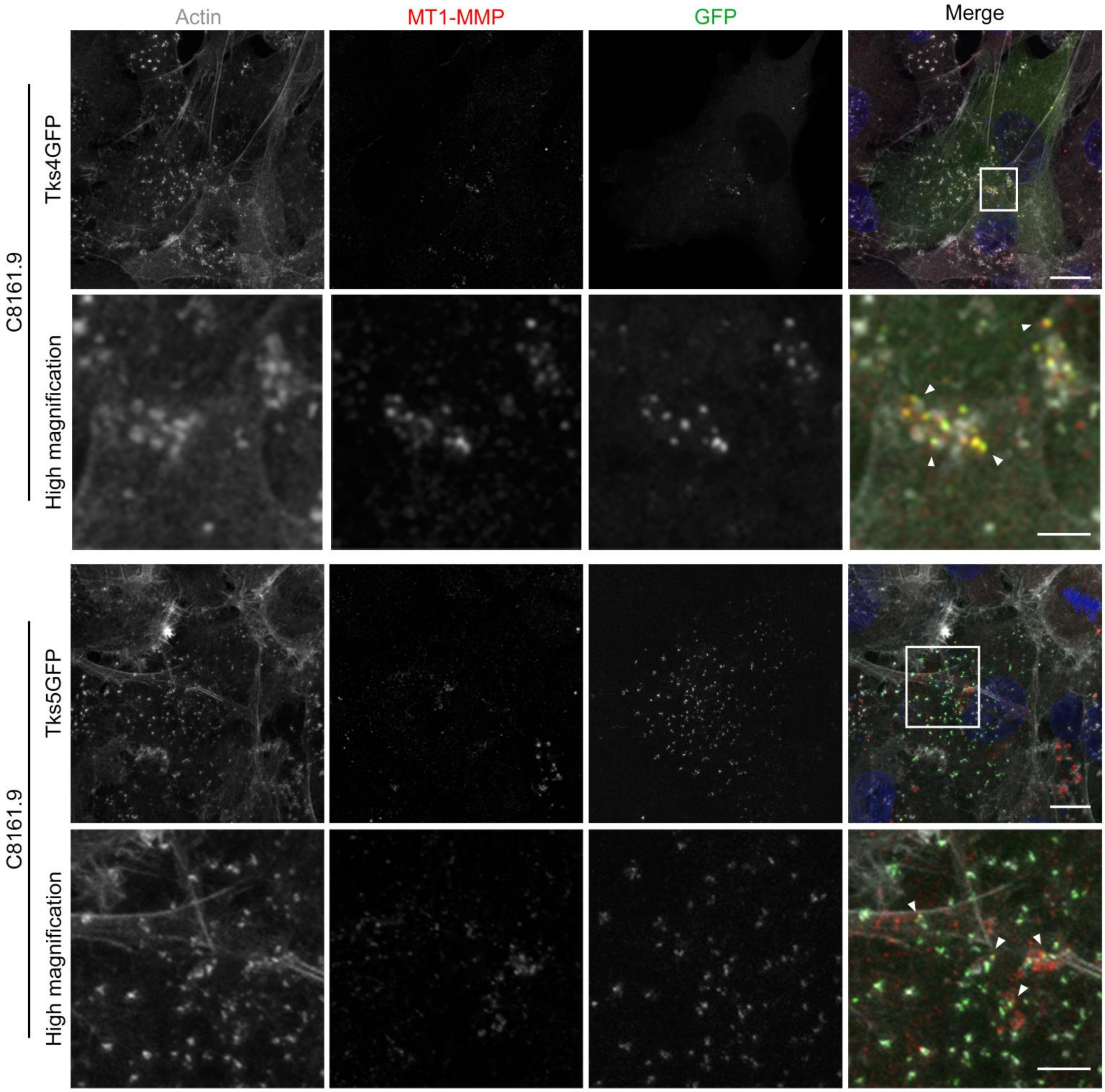

Supplemental Figure 7. Statistical analyses.
Area under the curve (AUC) analysis was performed for all 2D, 3D, and tumor growth assays. Individual growth curves were measured and the AUC function in GraphPad Prism was used to calculate the AUC. Student's t test was used to test significance. (A) AUC analysis for Figure 1F. (B) AUC analysis for Figure 4E. (C) AUC analysis for Figure 5B. (D) AUC analysis for Supplementary Figure 4E. (E) AUC analysis for Supplementary Figure 5. *p<0.005, **p<0.0005.
